# Decreased Frequency of Mental Workload-Induced Subjective Hot Flashes Through Gum Massage: An Open-Label, Self-Controlled Crossover Trial

**DOI:** 10.1089/whr.2021.0094

**Published:** 2022-03-08

**Authors:** Kanako Ichinose, Riho Tateyama-Makino, Asami Miyajima, Satoru Morishita, Taku Iwamoto, Yukio Yamamoto

**Affiliations:** Research & Development Headquarters, Lion Corporation, Edogawa-ku, Tokyo, Japan.

**Keywords:** hot flash, gum massage, somatosensory, oral cavity, mental workload, sympathetic nerve system

## Abstract

**Objective:**

Hot flashes, a symptom of menopause, can decrease women's quality of life. Sympathetic nervous system activation has been identified as an important factor in the occurrence of hot flashes. Given that somatosensory stimulation of the oral cavity can affect autonomic nervous activity, we aimed to investigate the possibility that somatosensory stimulation of the gums (*i.e.*, gum massage) could improve hot flashes.

**Materials and Methods:**

Nineteen women experiencing at least one hot flash per day were instructed to perform a gum massage on themselves before undertaking mental workload, using arithmetic task, and the frequency of hot flashes experienced during this task was measured. Changes in autonomic nervous activity were assessed based on heart rate variability.

**Results:**

Massage conditions promoted a significantly lower arithmetic task-induced hot flash frequency compared with nonmassage conditions (*p* < 0.05). During gum massage, the ratio between low and high frequency (LF/HF) values decreased significantly under massage conditions compared with nonmassage conditions (*p* < 0.01). During the arithmetic task, the gum massage-induced reduction in LF/HF, which changed from baseline, was significantly correlated with the gum massage-induced reduction in hot flash frequency.

**Conclusions:**

The results of this study indicate that gum massage can reduce the subjective frequency of hot flashes over a certain period under mental workload. Our study also indicates that gum massage can potentially decrease sympathetic nerve activity, which is known to be involved in the occurrence of hot flashes.

Clinical Trial Registration number 328 (the institutional review board of Lion Corporation).

## Introduction

Around the time of menopause, many women often develop uncomfortable menopausal symptoms, the most common of which is hot flashes that occur in 50%–80% of women undergoing menopause worldwide.^1,2^ Hot flashes, a vasomotor symptom, have generally been reported as feelings of intense warmth along with sweating and flushing in the face, neck, and chest, which usually last for 1–5 minutes, with some lasting as long as an hour.^3^ The median duration of symptoms is ∼4 years, with some lasting for as long as 20 years.^3^ Hot flashes can also cause mood disturbances and affect work productivity and quality of life.^1^

Menopausal women experience ovarian dysfunction, which consequently reduces estrogen levels.^1,4^ The brain's response to this hormonal change has been thought to cause thermoregulatory and vascular dysfunction, resulting in the occurrence of hot flashes.^1^ Hormone therapy has been widely regarded as a treatment method for hot flashes.^4^ In addition, vasomotor symptoms, including hot flashes, have been reported to be associated with changes in cardiovascular autonomic regulation in women.^3^ For instance, reports have shown that women with menopausal symptoms had a significantly higher low and high frequency (LF/HF) ratio, which is generally used as an indicator of sympathetic nerve activity,^5,6^ compared with those without menopausal symptoms.^7^

Moreover, the LF/HF ratio appeared to be further elevated among women whose hot flashes were higher than moderate.^7^ Low et al. reported that sympathetic cholinergic nerve activity is also increased during hot flashes.^8^ Previous findings also suggest that sympathetic nervous system activation is an important factor in the occurrence of hot flashes.^9–11^ Stellate ganglion block (SGB) is also another existing hot flash treatment method.^12,13^ Lipov et al. hypothesized that the SGB provides relief from hot flashes by interrupting connections between the central and sympathetic nervous systems.^14^

The LF/HF ratio is increased by daily stress^15,16^ and performing mental workload exercises such as arithmetic tasks and speech.^17^ Some previous study also suggest that mental stressors associated with increased hot flash^18,19^ and that mental workload was a risk factor for hot flashes.^20^ Considering this background, aside from estrogen replacement, suppressing sympathetic nerve activity is important for improving hot flashes.

At present, increased interest has been directed toward nonhormone therapy (complementary medicine),^21^ with reports suggesting that supplements with estrogen-like substances^22^ and herbal medicine^21^ improve hot flashes. Moreover, studies have suggested the efficacy of aromatherapy,^23^ foot reflexology,^24^ yoga,^25^ and exercise^26^ against hot flashes. Another study found that foot reflexology^27^ and aromatherapy^28^ can affect the autonomic nervous activity. While various hot flash treatments exist, exploring new treatments that suit diverse individual circumstances and lifestyles is imperative.

Previous studies have found that somatosensory stimulation of the oral cavity affected autonomic nervous activity. For instance, studies showed that chewing^29^ and compressing food^30^ may increase autonomic nervous system activity. Another study found that wiping the oral mucosa with a sponge brush may increase sympathetic nerve activity in elderly individuals requiring long-term care.^31^

Further evidence revealed that oral care with a toothbrush by a professional, which provides somatosensory stimulation to the tooth and gums, affected the central nervous system, subsequently initiating changes in parasympathetic nervous activities among healthy individuals.^32^ Therefore, previous findings suggest that somatosensory stimulation to the oral cavity modulates the autonomic nervous system.

Considering the presented background, we surmised that somatosensory stimulation to the gums (*i.e.*, gum massage) could improve hot flashes by modulating the autonomic nervous system. Therefore, we conducted this pilot, open-label, self-controlled crossover trial to identify the effects of gum massage on subjective hot flash frequency and severity.

## Materials and Methods

### Study design

This study was approved by the Institutional Review Board of Lion Corporation and conducted according to the guidelines of the Declaration of Helsinki and ethical guidelines for epidemiology research authorized by the Japanese government.

**FIG. 1.**
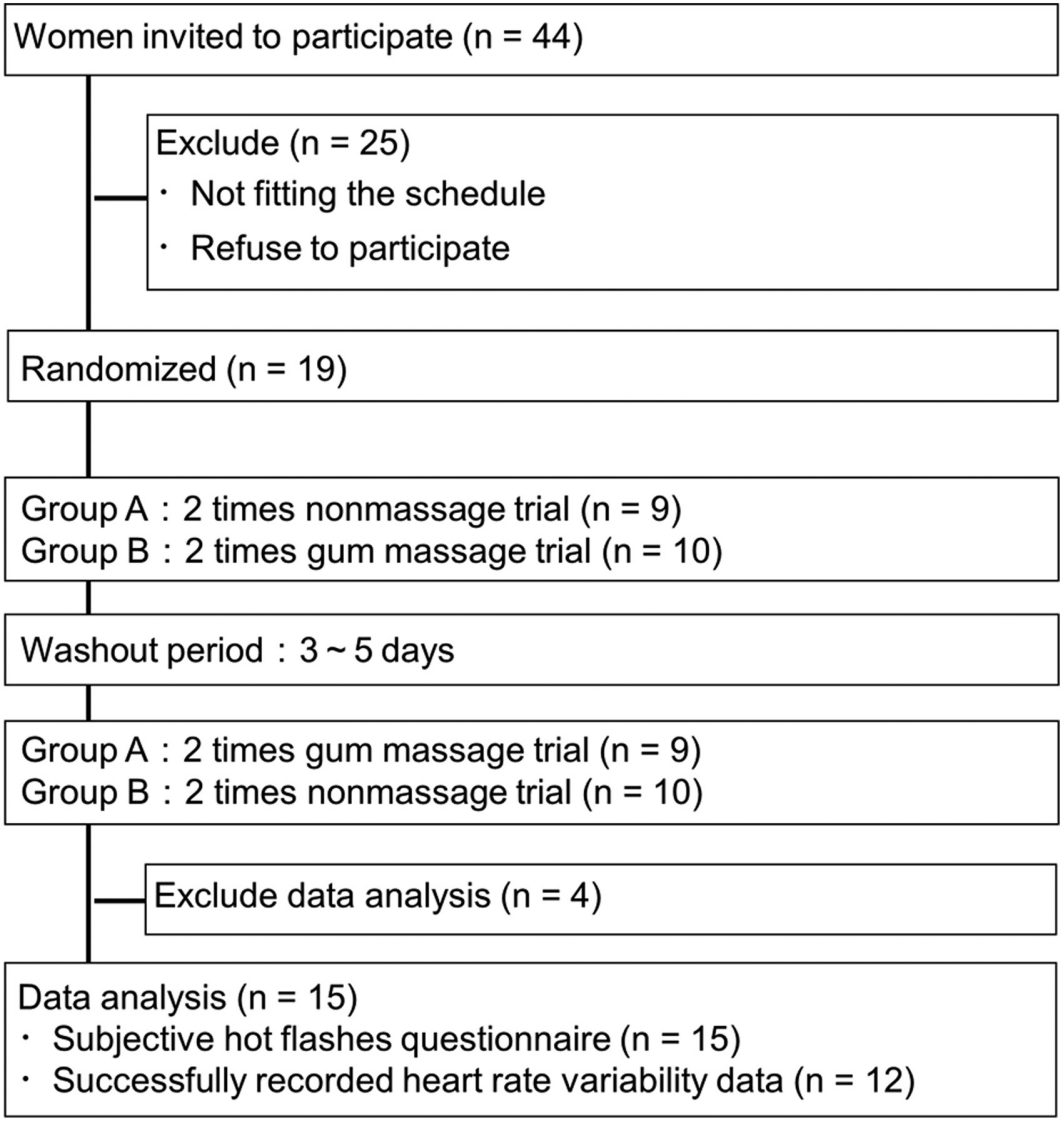
Flow chart of participation and study design. The flow chart shows the study design of the clinical trial. We randomly divided 19 participants, who satisfied the criteria, into two groups, namely groups A and B.

**FIG. 2.**
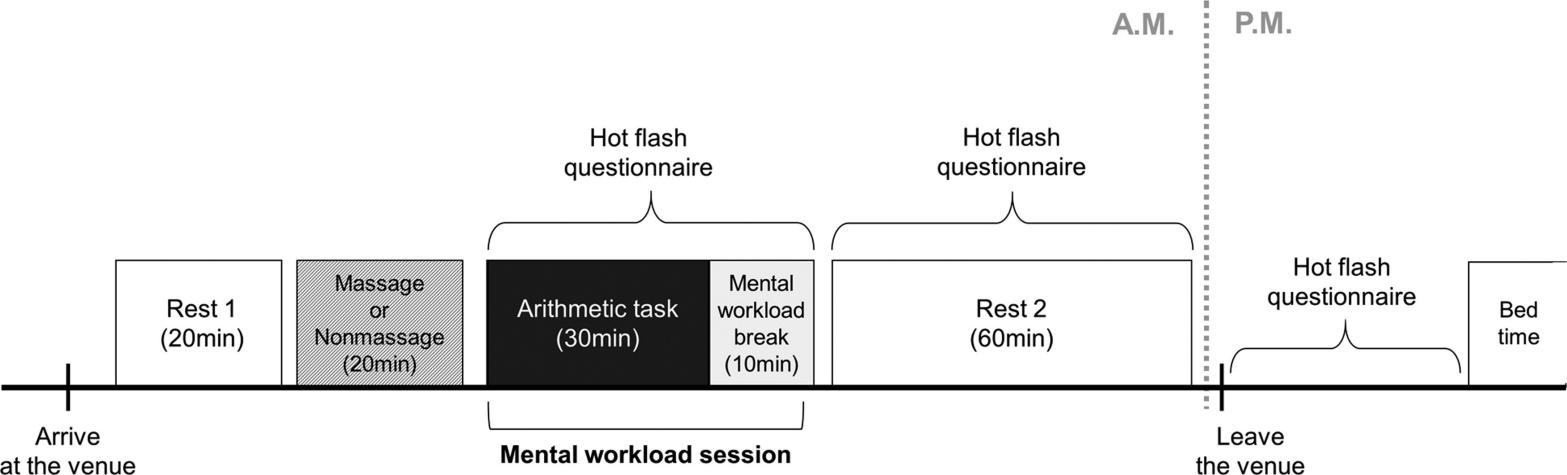
Experimental protocol of this study. Our trial was conducted using the following procedural flow: Rest 1, massage or nonmassage, mental workload (arithmetic task+mental workload break), and Rest 2. The subjective hot flash frequency was counted during arithmetic task, Rest 2, and afternoon. Heart rate variability was measured from Rest 1 to Rest 2. Arithmetic task and Mental workload break were done for 30 and 10 minutes, shown by the closed box (■) and gray color box, respectively. Massage or Nonmassage was done for 20 minutes, shown by the hatched box (▨). Rest is shown by the open box (□).

This open-label, self-controlled, crossover trial was conducted for a total of 4 days (gum massage trial twice and nonmassage trial twice) within a 2-week period. Participants were allocated to either group A or B based on the days they can participate (Fig. 1). Trials were conducted in the morning at Hirai Research Institute of Lion Corporation using the following procedural flow (Fig. 2): Rest 1 (20 minutes), massage or nonmassage (20 minutes), mental workload (arithmetic task and mental workload break, 40 minutes), and Rest 2 (60 minutes).

Subjective hot flashes occurring between mental workload and Rest 2 were then counted. Heart rate variability (HRV) was measured from Rest 1 to Rest 2. In the afternoon, participants counted the subjective hot flash frequency until bedtime in the living environment. The afternoon session lasted ∼10 hours and was spent without any specific stress on the participants.

### Participants

Menopausal Japanese women 40–60 years of age with stable menopause symptoms were recruited from 8000 volunteers registered at a research company (PLUG, Inc., Tokyo, Japan). Thereafter, 44 candidate participants were selected based on the inclusion criterion as the occurrence of hot flashes once per day. The exclusion criteria were based on a simple menopause index^33^ of ≥81 points,^34^ and with the ability to attend the explanation meeting and visit the clinical trial location (Fig. 1 and Table 1).

**Table 1.** Inclusion and Exclusion Criteria

Inclusion criteria	Exclusion criteria
Women between 40 and 60 years of age	Having severe menopausal symptoms requiring medical treatment. Menopause index >81
Having hot flash symptoms with sweating	Having hormone replacement therapy supplements during this study period and/or in the past
Frequency of hot flashes ≧1 time/day	Having chronic or mental illness
	Taking any of the following over-the-counter medications and/or supplements during this study period Improvement of menopausal symptoms Containing ingredients like female hormones Containing ingredients that affect the cardiovascular system Containing ingredients that affect the autonomic nervous system activity, sleep, fatigue, and stress
	Acupuncture, massage, and aromatherapy or relief of menopausal symptoms during the study period
	Smoking during the study period
	Having oral problems (inflammation of the gums, severe periodontal disease, temporomandibular disease, etc.)

Suitability for each criterion was determined based on self-reports from monitors through questionnaires. Among them, 19 participants who agreed to participate were selected (average age ± standard error [SE] = 52.2 ± 0.8 years). All participants provided written informed consent as required for enrollment and did not drop out from this study. Four participants were excluded from data analysis due to a cold during the study period (one participant), no hot flashes during the study period (one participant), and being able to measure only once for each of the massage and nonmassage conditions (two participants) (Fig. 1).

### Gum massage

Participants were advised to massage their maxillary gums (alveolar mucosa) with their own index fingers. The massage was performed with a pressure of ∼100 g. Before the start of a trial, all participants practiced pushing 100 g of pressure using an electronic scale. The subjects were then briefed on the massage method and they underwent a practice session for several minutes. Before massage or nonmassage session, the finger pressure of 100 g was checked again with an electronic scale. Starting from the maxillary gums on the left side of the body, participants moved their index fingers from the back teeth to the front teeth at a speed of ∼1 cm/s for 30 seconds.

After repeating this twice (1 minute), the maxillary gums on the right side of the body were massaged in similar manner. Completing the left and right sides was considered one set, with the participants performing 10 sets (20 minutes) in total.

### Inducing hot flashes through the mental workload

Hot flashes were induced using a method modified from a previous study by Park et al.^20^ During the task session, an arithmetic task was provided on a tablet PC screen using a mobile application (Brain Training-Ones Digit Calculation, a free application by Satoshi Nagamine). The participants were instructed to calculate two numbers next to each other and answer one digit. The 5-minute calculation was taken as one test. After repeating this test six times (30 minutes), the participants took a break for 10 minutes.

### Measuring subjective hot flash frequency and severity

The subjective hot flash frequency was determined using a method modified from a previous study by Guttuso et al.^35^ The participants were requested to report the intensity of the hot flash after each session during the arithmetic task (every 5 minutes), break phase of the mental workload (once only), and Rest 2 (every 10 minutes) based on the following criteria: mild (sensation of heat without sweating), moderate (sensation of heat with sweating), severe (sensation of heat with sweating causing disruption of current activity), and no hot flash.^35^

The subjective hot flash frequency for each session (the No. of terms) was then calculated. The severity of the hot flashes was evaluated using “heating only” and “with sweating” and was calculated based on the frequency of mild and total frequency of moderate and severe hot flashes. In the afternoon session, another diary was used in which participants wrote down the intensity, start time, and duration each hot flash felt.

### Heart rate variability

Data regarding HRV were recorded using a heart rate monitor (WHS-1; Union Tool Co., Tokyo, Japan) throughout the experimental period and analyzed using software (RRI Analyzer; Union Tool Co.). In the analysis of HRV, heart rate R-R interval (RRI) data were Fourier transformed, and the power spectrum was calculated in frequency units. The total amount of the power spectrum at LF(0.04–0.14 Hz) was considered mainly reflecting sympathetic activity, whereas that of the power spectrum at HF(0.14–0.4 Hz) reflected parasympathetic activity.^5,36–38^ LF/HF and LF/(LF + HF), calculated from HRV, are used as indicators of sympathetic nerve activity.^5,28,39^

We calculated the HRV at each session using methods described previously.^17,39^ LF, HF, LF/HF, and LF/(LF + HF) were used to calculate the 30-second epoch (calculation time; 120 seconds). The average value of epochs for each session, namely Rest 1, massage or nonmassage, arithmetic task phase, break phase during the mental workload, and Rest 2, was calculated. The change values from Rest 1 for each index, LF, HF, LF/HF, and LF/(LF + HF), were also calculated^28,40–42^; these change values are denoted as ΔLF, ΔHF, ΔLF/HF, and ΔLF/(LF + HF), respectively. HRV data for three participants were excluded from statistical analysis, given that many of their RRI values exceeded the upper (2000 ms) or lower (300 ms) limit (Fig. 1).

### Correlation analysis

We performed correlation analysis using the difference between the results of the massage and nonmassage conditions (difference value between conditions: nonmassage conditions−massage conditions). For hot flash frequency, we used the difference between the results of the massage and nonmassage conditions during the arithmetic task for each participant. For the HRV values, the value of the difference between the results of the massage and nonmassage conditions during the arithmetic task for each participant was calculated and used in the correlation analysis.

### Statistical analysis

All data were expressed as mean value ± SE. To compare differences during hot flashes between the massage and nonmassage conditions, data were analyzed using a paired and nonparametrical test (Wilcoxon signed-rank test) and analysis of covariance (ANCOVA). Spearman's rank correlation analysis was used for correlation analysis between different indicators. All analyses were conducted using JMP version 13.2 (SAS Institute Japan Co., Tokyo, Japan) with *p* < 0.05 indicating statistical significance.

## Results

### Effects of gum massage on subjective hot flash frequency and severity

Our analysis showed that the massage conditions promoted significantly fewer arithmetic task-induced hot flashes than the nonmassage conditions (*p* = 0.039) (Table 2). No significant difference in the frequency of subjective hot flashes—described as “heating only” or “with sweating”—was found between the massage and nonmassage conditions (Table 2). However, the comparison of the massage and nonmassage conditions in terms of “heating only” frequency on the arithmetic task resulted in a *p* value of 0.088. No significant difference was observed for the number of hot flashes during task break, Rest 2, and afternoon sessions (Table 2).

**Table 2.** Comparison of Hot Flash Frequency in the Massage and Nonmassage Conditions

	Mental arithmetic task			Mental arithmetic task break			Rest 2			Afternoon		
Self-reported hot flash frequency per session												
	Nonmassage	Massage	*p*	Nonmassage	Massage	*p*	Nonmassage	Massage	*p*	Nonmassage	Massage	*p*
All	3.03 ± 0.57	2.37 ± 0.59	0.039	0.25 ± 0.08	0.32 ± 0.10	0.766	0.77 ± 0.26	0.90 ± 0.29	0.633	1.93 ± 0.30	1.70 ± 0.34	0.283
Heating only	2.53 ± 0.50	1.83 ± 0.47	0.088	0.25 ± 0.09	0.25 ± 0.09	1.000	0.70 ± 0.23	0.90 ± 0.29	0.457	1.27 ± 0.25	1.23 ± 0.24	0.938
With sweating	0.50 ± 0.28	0.53 ± 0.31	1.000	0.00 ± 0.00	0.07 ± 0.05	0.500	0.07 ± 0.05	0.00 ± 0.00	0.500	0.66 ± 0.27	0.47 ± 0.24	0.531

### Effects of gum massage on HRV and the relationship between HRV and subjective hot flash frequency

We investigated the effects of gum massage on the autonomic nervous system using HRV. No significant difference in HRV was noted between the two conditions during the Rest 1 session (Table 3). During the massage or nonmassage session, LF, LF/HF, and LF/(LF+HF) values, including the relative value from Rest 1 session [ΔLF, ΔLF/HF, and ΔLF/(LF + HF)], decreased significantly in the massage condition compared with those in the nonmassage condition (*p* < 0.01) (Table 3). ANCOVA test using LF, LF/HF, LF/(LF + HF), ΔLF, ΔLF/HF, and ΔLF/(LF + HF) with Rest 1 value as the covariate also showed a significant difference between the massage and nonmassage conditions (Supplementary Table S1).

**Table 3.** Comparison of Autonomic Nervous Activity in the Massage and Nonmassage Conditions

	HRV values			HRV values changed from Rest 1		
	Nonmassage	Massage	*p*	Nonmassage	Massage	*p*
Rest 1						
LF	460.06 ± 43.41	434.90 ± 46.26	0.622			
HF	412.84 ± 82.80	370.09 ± 76.57	0.677			
LF/HF	1.79 ± 0.30	2.36 ± 0.53	0.151			
LF/(LF+HF)	0.55 ± 0.04	0.57 ± 0.04	0.519			
Massage or nonmassage						
LF	477.82 ± 41.76	251.35 ± 19.07	<0.001	17.76 ± 24.25	−183.55 ± 37.30	<0.001
HF	407.82 ± 87.18	354.73 ± 63.13	0.301	−5.02 ± 14.19	−15.36 ± 36.83	0.677
LF/HF	2.04 ± 0.42	1.25 ± 0.23	0.009	0.25 ± 0.16	−1.11 ± 0.34	<0.001
LF/(LF+HF)	0.56 ± 0.04	0.45 ± 0.04	0.003	0.01 ± 0.01	−0.12 ± 0.02	<0.001
Mental arithmetic task						
LF	334.61 ± 35.93	286.12 ± 28.49	0.266	−125.46 ± 37.03	−148.78 ± 34.24	1.000
HF	338.50 ± 89.30	273.10 ± 63.00	0.470	−74.34 ± 33.28	−97.00 ± 46.21	0.424
LF/HF	1.59 ± 0.21	1.74 ± 0.27	0.424	−0.20 ± 0.17	−0.61 ± 0.31	0.092
LF/(LF+HF)	0.53 ± 0.04	0.54 ± 0.04	0.677	−0.02 ± 0.02	−0.03 ± 0.02	0.733
Rest 2						
LF	560.08 ± 39.00	474.90 ± 37.93	0.052	100.02 ± 36.25	39.41 ± 16.83	0.064
HF	390.04 ± 66.75	361.40 ± 61.90	0.733	−22.81 ± 29.82	−8.96 ± 43.23	0.677
LF/HF	2.24 ± 0.41	2.44 ± 0.52	0.380	0.45 ± 0.24	0.08 ± 0.14	0.052
LF/(LF+HF)	0.58 ± 0.04	0.58 ± 0.04	0.570	0.04 ± 0.02	0.01 ± 0.02	0.109

HF, high frequency; HRV, heart rate variability; LF, low frequency.

No significant difference in HRV was observed between the two conditions during the arithmetic task and the Rest 2 session (Table 3). In addition, no significant difference in ΔLF, ΔLF/HF, and ΔLF/(LF + HF) was observed between the massage and nonmassage conditions during the arithmetic task and Rest 2 sessions (Table 3).

Gum massage significantly reduced the frequency of subjective hot flashes during the arithmetic task session (Table 2), but there was no significant difference in sympathetic nerve activity (LF/HF and LF/[LF + HF] value) between the massage and nonmassage conditions during the arithmetic task session (Table 3). Nevertheless, because a decrease in sympathetic nerve activity was noted during the massage or nonmassage session (Table 3), we hypothesized the presence of a correlation between the change in the number of subjective hot flashes by gum massage during the arithmetic task session and that of sympathetic nerve activity during this session.

Subsequently, we performed a correlation analysis between the number of hot flashes and sympathetic nerve parameters [LF/HF and LF/(LF+HF)] at the arithmetic task session to investigate the relationship between subjective hot flash reduction and autonomic nervous activity. Accordingly, no significant correlation was noted between the difference value between the conditions of subjective hot flash frequency and that of LF/HF or LF/(LF+HF) during the arithmetic task (Table 4). However, a significant correlation was found between the difference value between the conditions of subjective hot flash frequency and that of ΔLF/HF (*ρ* = 0.7014, *p* < 0.05) or ΔLF/(LF + HF) (*ρ* = 0.8183, *p* < 0.01) (Table 4).

**Table 4.** Correlation Analysis Between Subjective Hot Flash Symptoms and Heart Rate Variability During the Arithmetic Task

	Data		Changes from Rest1	
	LF/HF (massage–nonmassage)	LF/(LF+HF) (massage–nonmassage)	LF/HF (massage–nonmassage)	LF/(LF+HF) (massage–nonmassage)
Hot flashes frequency per session (massage–nonmassage)	*ρ* = −0.183 *p* = 0.5699	*ρ* = −0.212 *p* = 0.5085	*ρ* = 0.701 *p* = 0.0110	*ρ* = 0.818 *p* = 0.0011

## Discussion

Controlling autonomic nerve activity (suppression of sympathetic nerves) has been suggested as an effective means for suppressing hot flashes. Therefore, this pilot study examined whether gum massage, which is thought to affect autonomic nervous activity, could reduce the occurrence of hot flashes. Notably, these research results suggest that gum massage improves the subjective frequency of hot flashes over a certain period under mental workload. To our knowledge, this has been the first study to demonstrate reduction in subjective hot flashes through gum massage.

Reports have also shown that sympathetic nerve activity increases during the occurrence of hot flashes and that some of hot flash treatments involve reducing sympathetic nerve activity. Both LF/HF and LF/(LF + HF) calculated from HRV are used as indicators of sympathetic nerve activity.^5,28,39^ Our results found that LF/HF and LF/(LF+HF) were significantly lower under massage conditions than under control conditions (Table 3). Given that a decrease in LF/HF and LF/(LF+HF) implies a suppression of sympathetic nerve activity,^28^ the aforementioned results indicate that the gum massage suppresses sympathetic nerve activity during the massage.

Although studies have shown that somatosensory stimulation of the oral cavity affects the autonomic nervous system,^29–32^ to our knowledge, this has been the first study to show that gum massage decreased sympathetic nerve activity and that such a reduction may reduce the frequency of subjective hot flashes. However, LF/HF and LF/(LF+HF) values did not change significantly during the arithmetic task, despite the actual decrease in the number of hot flashes.

However, based on the correlation analysis between the amount of change in sympathetic nerve activity and the frequency of hot flashes, our results suggest a correlation between changes in autonomic nerve activity and hot flashes due to gum massage (Table 4). For example, a positive correlation between these changes indicates that sympathetic nerve activity decreased more with a larger decrease in the frequency of hot flashes during the arithmetic task session. In other words, if a positive correlation was found, it is possible that those who experienced a reduced number of subjective hot flashes during the arithmetic task session by gum massage had reduced sympathetic nerve activity during this session.

As a result, reductions of ΔLF/HF and ΔLF/(LF + HF) were significantly correlated with the reduction in subjective hot flash frequency following gum massage (Table 4). Because the absolute value of HRV differs widely among individuals and tends to change, a significant correlation was found only in ΔLF/HF and ΔLF(LF + HF), which excludes the effect of the initial value. Although further verification is required in studies with more measurements and participants, it is possible that those who experienced suppression in subjective hot flash frequency induced by the arithmetic task following gum massage tended to have suppressed sympathetic nerve activity during the arithmetic task.

This study found that gum massage reduced the number of hot flashes (Table 2). In contrast, no difference in the frequency of subjective “with sweating” hot flashes was observed between both conditions, despite having recruited subjects who usually experienced hot flashes with sweating (Table 1). However, more than half (53.3%) did not experience hot flashes with sweating during the arithmetic task. Our study was conducted at 25°C, given the limitations in temperature control at the testing facility, whereas Park et al.'s study, on which the test design of this study was based, conducted their study at 28°C.^20^ In fact, Park et al.'s study showed that hot flashes with sweating occurred 1.625 times/h·person during the arithmetic task.^20^

Despite the differences in measurement methods of hot flash frequency between Park et al.'s study and our own, our results showed that hot flashes with sweating occurred 1.00 times/h·person during the arithmetic task, with the frequency of hot flashes with sweating being lower than expected. Evidence has shown that small elevations in core body temperature trigger hot flashes^3^ and that core body temperature rises due to sudden changes in temperature (room temperature, etc.)^43^ and body movements (exercise, etc.).^3^ This difference in room temperature may have affected the frequency of subjective “with sweating” hot flashes.

As such, the design of this study may not be suitable for measuring hot flashes accompanied by subjective sweating. Therefore, when confirming the effects of gum massage on the frequency of hot flashes with sweating, studies need to be conducted under higher room temperature conditions. Given that participants developed hot flashes with sweating during their daily life (Table 1), studies that mimic daily life through the absence of behavioral restrictions may also be appropriate.

In this study, gum massage decreased arithmetic task-induced subjective hot flash frequency. Since the arithmetic task is one of the mental workloads and also become a stressor, it was suggested that gum massage could have a reduction effect on stress-induced hot flashes. However, hot flashes can also occur unexpectedly without stress. Given that this study was conducted under the specific arithmetic task conditions, further studies should be conducted to evaluate the efficacy of gum massage in reducing hot flashes that occur in daily life.

This study suggested that a decrease in sympathetic nerve activity through gum massage may contribute toward reduced frequency of subjective hot flashes. However, the mechanism of hot flash suppression by gum massage is still largely unknown. Therefore, further studies are warranted to elucidate it. For instance, to clarify the contribution of the gingival somatosensory system toward the reduction in subjective hot flash frequency, comparing the suppressive effects of a gum massage and pseudo-gingival massage without any somatosensory input from the gingiva is necessary.

In addition, given that somatosensory input from the oral cavity projects to the primary somatosensory cortex of the cerebral cortex^44^ and that autonomic nervous activity is controlled by the hypothalamus,^45^ it is also necessary to examine changes in brain activity within these areas during gum massage to clarify the mechanism by which gum massage suppresses subjective hot flashes.

## Conclusions

The results of this study suggest that gum massage improves the subjective frequency of hot flashes over a certain period under mental workload. Our study also indicates that gum massage has the potential to decrease sympathetic nerve activity, which is known to be involved in the occurrence of hot flashes. Therefore, gum massage can aid in the development of a new nonhormonal therapy for hot flashes under stress. For instance, the massage method presented herein could potentially be incorporated into daily oral care, such as brushing behavior.

## Supplementary Material

Supplemental data

## Abbreviations Used

ANCOVA: analysis of covariance
HRV: heart rate variability
LF/HF: low and high frequency
RRI: R-R interval
SE: standard error
SGB: Stellate ganglion block
