# Supplementary material for: Decreased Frequency of Mental Workload-Induced Subjective Hot Flashes Through Gum Massage: An Open-Label, Self-Controlled Crossover Trial
Source: Womens Health Rep (New Rochelle). 2022 Mar 8;3(1):335–43. doi: 10.1089/whr.2021.0094 (PMC8994437; doi:10.1089/whr.2021.0094)
Supplement: Supplemental data [file Suppl_TableS1.DOCX]

Supplemental Table 1

|  | HRV values | | | | | | HRV values changed from Rest 1 | | | | | |
| --- | --- | --- | --- | --- | --- | --- | --- | --- | --- | --- | --- | --- |
|  | ANCOVA　 condition | | ANCOVA　 Rest 1 value | | ANCOVA　 condition × Rest 1  value | | ANCOVA　 condition | | ANCOVA　 Rest 1 value | | ANCOVA　 condition × Rest 1  value | |
|  | F value | P value | F value | P value | F value | P value | F value | P value | F value | P value | F value | P value |
| Massage or Non-massage |  |  |  |  |  |  |  |  |  |  |  |  |
| LF | 55.624 | <0.001 | 30.962 | <0.001 | 8.131 | <0.001 | 41.696 | <0.001 | 19.112 | <0.001 | 1.41 | 0.249 |
| HF | 0.194 | 0.664 | 178.578 | <0.001 | 5.712 | 0.029 | 0.194 | 0.664 | 3.197 | 0.089 | 5.712 | 0.027 |
| LF/HF | 53.903 | <0.001 | 154.989 | <0.001 | 46.536 | <0.001 | 53.903 | <0.001 | 4.143 | 0.055 | 46.536 | <0.001 |
| LF/(LF + HF) | 37.572 | <0.001 | 134.991 | <0.001 | 1.926 | 0.181 | 37.572 | <0.001 | 0.155 | 0.698 | 1.926 | 0.181 |
